# Solones, Solo Reproduction and Vice

**DOI:** 10.1007/s41649-025-00381-1

**Published:** 2025-08-27

**Authors:** Anantharaman Muralidharan, Julian Savulescu

**Affiliations:** 1 Centre for Biomedical Ethics, Yong Loo Lin School of Medicine, National University of Singapore, Singapore; 2 Murdoch Children’s Research Institute, Melbourne, Australia; 3 Department of Philosophy, University of Oxford, Oxford, UK

**Keywords:** In vitro gametogenesis, Solo reproduction, Vice, Cloning

## Abstract

In vitro gametogenesis (IVG) is a technology that allows the creation of gametes from stem cells. Given that IVG makes possible the production of cross-sex gametes, IVG, if successful, can, among other uses, allow a single person to solo reproduce (i.e. have children without a sperm or egg donor). This would involve using IVG to produce one cross-sex gamete and fusing that gamete with a same-sex gamete which is either artificially or naturally derived from the same person. Because solo reproduction is a highly experimental, artificial and asexual method of reproduction, it might be comparable in many ways with reproductive cloning. One of the arguments that has been made against cloning and, by extension, solo reproduction is that it expresses and encourages vices like hubris and narcissism. In this paper, we argue that the vice argument is insufficient to justify a ban on solo reproduction. This is because the mere fact that an act is vicious does not give anyone any kind of enforceable claim on us to not act viciously.

## Introduction

Recent improvements in epigenetic reprogramming technologies (Murase et al. 2024) have brought the possibility of in vitro gametogenesis (IVG) closer to reality. IVG is a technology which allows gametes to be produced from a person’s somatic cells. It promises to be a technology that can address forms of infertility that current in vitro fertilisation (IVF) techniques are unable to. After all, IVF still requires a sperm and egg be provided by the biological parents. Insofar as certain forms of infertility deprive some persons of the capacity to produce gametes, IVG can help such persons. IVG also promises to allow same sex couples to have children who are approximately equally genetically related to both parents.

The same technology which can help couples who are infertile for medical or social reasons can also allow a single individual to reproduce entirely by themselves (or at most with the help of a surrogate). While solo reproduction in this way bears some superficial similarity to cloning, laws which are explicitly aimed at restricting reproductive cloning often do not restrict solo reproduction using IVG. Yet we might foresee that many of the same people who objected to reproductive cloning would object to solo reproduction too even if the technology were to achieve a parity in terms of physiological risk comparable with other accepted reproductive technologies. This opens up the question as to whether solo reproduction *should* be legally restricted. While there are many objections to cloning and, by extension, solo reproduction, most of them have already received much attention. One argument, which has received comparatively little attention, is the vice argument, according to which solo reproduction (and cloning) is wrong and hence should be banned because it expresses and encourages vices like hubris and narcissism. In this paper, we will argue that, if sufficiently safe and reliable, solo reproduction should be permitted because merely expressing or encouraging certain vices is not any sort of reason to ban an act.

Our strategy in this paper is as follows: In the “Solo Reproduction, Cloning and the Law” section, we will explain what solo reproduction is and distinguish it from cloning. We will also discuss the legal background and show how laws aimed at addressing cloning do not address solo reproduction with IVG. In the “Other Arguments for the Prohibition of Solo Reproduction” section, we survey arguments for banning solo reproduction. This includes arguments about distress, compensatory justice, dignity and the purpose of human sexuality. We argue briefly either that these arguments are unsuccessful or that they involve imposing a religious or otherwise equally controversial account of the good life on people. In the “Vice, Virtue and the Duty to Do Virtuous Things” section, we focus on the vice argument. We discuss different conceptions of why the virtues might be valuable and show that there is no plausible account which requires anyone to avoid all actions that express or encourage vices. In the “Conclusion” section, we conclude by considering the implications for using IVG in less contentious cases as well as for laws on reproductive cloning.

## Solo Reproduction, Cloning and the Law

Solo reproduction refers to a process by which a child is conceived by the fusion of two gametes, both of which originate from the same person. This process would be made possible by IVG technology. IVG is a process by which gametes are created from embryonic stem cells or pluripotent stem cells induced from somatic cells (Notini et al. 2020; Saitou and Hayashi 2021; Murase et al. 2024). These stem cells can be stimulated to become primordial germ cell–like cells (PGCLCs). Like primordial germ cells, these cells will develop, eventually, into sperm and egg cells. In principle, if the technology is successful, scientists would be able to control whether the PGCLCs develop into sperms or eggs. This means that through this technology, a genetic male could give rise to eggs, and a genetic female could give rise to sperm. For simplicity of expression, let us call the person who gives rise to the sperm, the “biological father”, and the person who gives rise to the egg, the “biological mother”. This terminology matches the way we conventionally attribute biological parenthood, namely the source of the sperm is the biological father and the source of the egg is the biological mother.

Since gametes can be generated from a person without them needing the requisite or, for that matter, any gonads, IVG allows people who are infertile because of their inability to produce any gametes to have children. The ability to generate cross-sex gametes also means that same-sex couples can have children who are roughly equally genetically related to both of them. The genetic mother would be slightly more related because they contribute mitochondrial DNA as well as nuclear DNA. IVG also likewise makes it possible for trans-women to become biological mothers and trans-men to become biological fathers. The particular use of IVG that is the focus of this paper is solo reproduction. Here, the same person is both biological mother and father of a particular child. This is done by using IVG to produce one cross-sex gamete and combining that with another gamete from the same individual. The other gamete can either be generated naturally or by IVG. For ease of expression, let us call the child that results from solo reproduction a “solone”.

Solones are different from clones in the following way. Clones are approximately genetically identical to their “parent”. Clones are only approximately and not perfectly identical because there is, inevitably, a small rate of random mutation. While some women would be able to use their own eggs and hence ensure that the mitochondrial DNA of the clone is approximately identical to her parent’s other people who cannot or are not willing to donate an egg will have to use a donor’s egg. Hence, their clones will have different mitochondrial DNA as well. Solones, on the other hand, are unlikely to be genetically identical. There is only a 75% chance^1^ that a solone will inherit at least one copy of any given gene from their parent.

There is one very big difference between solones and clones. On average, each person carries 1–2 recessive mutations for severe disease (Gao et al. 2015). As inbreeding increases, the risk of having a child with a severe recessive genetic condition radically increases. For example, in a study conducted in Prague, the chance for the offspring of brother-sister or father daughter incest having a birth-defect (52%) is about six times as high as the chance would be for their half-siblings who are not the product of incest (8.7%) (Seemanová 1971). Given that genetic load can vary between subgroups,^2^ the risk of severe birth defect for a solone may be 24% or higher^3^ if left up to chance. Thus, in one way, solo reproduction is much more unsafe than cloning. However, there are now ways of reducing this risk through genetic testing and ultimately, if it were safe, gene editing. For instance, even if the risk of severe genetic abnormalities was 75%, an embryo without severe genetic abnormalities could be found so long as enough embryos were created. Unlike IVF where the number of embryos is limited by how many eggs that can be harvested (often painfully) from the mother, IVF could painlessly generate as many embryos as needed from induced pluripotent stem cells. Solo reproduction by IVG would require either prenatal testing or preimplantation genetic testing and whole-genome sequencing to assess whether a severe genetic disorder was present. With preimplantation genetic testing, the risk may be greatly reduced.

Despite these differences, solo reproduction is, in many ways, morally similar to reproductive cloning. For one, cloning is one method by which a person may reproduce without involving anyone else’s genetic material. Secondly, like cloning, some people might see it as a particularly “unnatural” way of reproducing. While, unlike cloning, it involves the union of a sperm and an egg, either or both gametes are not generated “naturally” (i.e. formed in the testes or ovaries). Critics of both may charge that solo reproduction and cloning involve a kind of promethean striving to exceed human limitations, that it evinces a desire for a specific genetic makeup for one’s offspring or that it involves a kind of narcissism (Malby 2002; Sandel 2005). Those who find cloning repulsive would likely have a similar, if not stronger, reaction to solo reproduction. That said, in most jurisdictions, they are treated very differently.

For instance, in places like the USA, Australia and Singapore, cloning is explicitly banned, but there are no statutory limitations on solo reproduction, despite its high risks. In these jurisdictions, the law permits the implantation and gestation of embryos for the purpose of reproduction so long as the embryos are the product of the fusion of a human egg and sperm. The US Human Cloning Prohibition Act of 2003 specifies that the restriction is placed on “reproduction not initiated by union of sperm and oocyte”. Likewise, the Australian Research Involving Human Embryos Act 2002 prohibits the use of embryos which are “created by a process other than the fertilisation of a human egg by a human sperm” (§10 A). Notably, while Australian law prohibits the creation of an embryo with genetic material from more than two persons, it does not prohibit the creation of an embryo with genetic material from only one person. According to Singapore’s Human Cloning and other Prohibited Practices Act 2004, a human embryo clone refers to “any human embryo that is a genetic copy of another living or dead human but does not include a human embryo created by the fertilisation of a human egg by human sperm”.

To be clear, there might be other legal restrictions on solo reproduction. US law, for instance, would forbid federal funding of IVG. The provision of assisted reproductive services in Singapore requires ministerial approval (HCSA 2023). Regulations that licence the clinics that provide such services licence them for specific services, and IVG is currently not listed among those services, in part because the technology is not fully mature yet. In the future when the technology becomes viable, the Director-General may, at his or her discretion, withhold approval of some or all applications of IVG technology.

Nevertheless, there is still a legal distinction between discretionary administrative barriers to IVG, on the one hand, and more stringent statutory limitations of IVG and solo reproduction on the other. Consider, for instance, the case of the UK where the Human Fertilisation and Embryology Act of 2008 permits only embryos made of sperm and eggs that have been extracted from testes and ovaries, respectively. These rather explicit provisions forbid, in principle, the use of IVG on humans. In Singapore, by contrast, there is no in principle barrier, only a de facto restriction. The Director General might, after all, be persuaded to act otherwise.

To be clear, in many of these jurisdictions, the failure to explicitly restrict IVG and solo reproduction is less a matter of deliberate policy and more a failure to anticipate IVG technology. With recent advances in epigenetic research bringing IVG and solo reproduction closer to reality, it has become timely to ask what our policy towards IVG should be in the event that IVG could be used to consistently produce a healthy child who is genetically related to only one parent.

## Other Arguments for the Prohibition of Solo Reproduction

A number of objections have been offered against legalising solo reproduction and IVG. Since we do not have the space to address all the arguments in full detail, we will first briefly address the objections which have been discussed elsewhere and show that these other objections fail. Subsequently, we will address what we call the vice objection.

### Distress, Justice and the Question of Default Entitlement

One argument against solo reproduction is that people experience less infertility-related distress for being unable to solo reproduce than for being unable to reproduce with their partner (Notini et al. 2020). Hence, or so the argument goes, there is little positive reason to allow people access to IVG for the purpose of solo reproduction.

One problem with this argument is that it presupposes that the default policy option is to deny access to some procedure and allow access only if there is sufficient positive reason for that access. However, this gets the normative burden of proof for regulations backwards. Coercive restrictions, such as bans on solo reproduction, are prima facie infringements on individual autonomy. As such, coercive restrictions require some positive, sufficiently strong justification (Haining et al. 2024). To be sure, we need not go so far as to presuppose something like Mill’s Harm principle or a public justification requirement here. We are merely presupposing that there must be some positive reason to coercively restrict people’s options. The absence of a positive reason for permitting access does not suffice for justifying a ban. Mere lack of benefit does not suffice to justify a ban, though it could be the basis of declining to offer any public subsidy or research and development support since there may be no public interest advanced through facilitating solo reproduction.

Notini and colleagues offer another argument that suffers from the same problem. They argue that compared to using IVG for same sex couples, people who intend to solo reproduce have less of a claim to use IVG technology. They argue that while singles, like same sex couples, are entitled to compensatory justice and hence owed access to assistive reproductive technologies, this does not include the use of IVG for solo reproduction. That is because singles can attain genetic parenthood (but not solo genetic parenthood) by other means, such as using donor gametes.

This argument presupposes firstly that the option of being the sole genetic parent is not valuable and secondly that restricting access to non-valuable options is not wrong. However, neither assumption seems obviously true. With regard to the second assumption, the presumption that the default stance is prohibition and that the value of an option needs to be established in order for access to be permitted is authoritarian. As with the distress argument, the assumption that what is authoritarian is the assumption that we do not need any positive reason to coercively restrict other people’s choices. The mere absence of specific reasons not to restrict is not enough. There must, at the minimum, be some positive reason to justify coercion.

With regards to the first assumption, there might be some reason to think that being the sole genetic parent is a valuable option. After all, many people consider being genetically related to one’s children to be valuable in and of itself. If there is some value in having a child who is only 50% related to oneself, it does not seem like an obvious mistake, even if highly unusual, to value having a child who is related to an even greater degree even more. Just as a couple may legitimately desire that they have a child without genetic input from unrelated others, a single parent’s desires to have a child without genetic input from those outside the family unit may be legitimate too (Suter 2016).

The broad lesson we can take from the discussion of these objections is that in order to justify a ban on solo reproduction, we must show that it is objectionable, in and of itself. Despite the details of the process differing, cloning, like solo reproduction, is an assisted reproductive technology (ART) by which a person may have offspring without involving anyone else’s genetic material. As such, some arguments against cloning have been extended to solo reproduction. There are also some other arguments which have not yet been extended which could be.

### Dignity and Human Sexuality

We can classify objections to cloning under four broad categories: concern for the biological wellbeing of the resultant child, dignitarian concerns, concerns about the teleology of human sexuality and concerns about whether cloning expresses vicious dispositions. The main focus of this paper is on the question of vice and will be discussed in the “Vice, Virtue and the Duty to Do Virtuous Things” section.

There are a number of reasons for focusing on vice. Firstly, concerns about the biological well-being of the resultant child are only of concern where IVG and preimplantation genetic screening technologies are not sufficiently reliable and that is beyond the scope of this paper. Secondly, concerns about dignity and the teleology of human sexuality have already been well-discussed in the literature.

To be sure, it would be reasonable to object to a new reproductive technology that had, say, a 50% chance of producing an abnormal child. It is not safe enough. However, in this paper, we are interested only in the case where the technology (with the use of whole-genome sequencing for preimplantation or prenatal testing) is at least as reliable as currently accepted techniques like IVF in giving rise to children without significant birth defects. This is because biological risk is better regulated by safety and efficacy regulations. If our only concern is biological risk to the solone, we should permit research that can reduce such those risks. Presumably, research could be less risky because it would first have to be shown to be consistently reliable with non-human animals, biomarkers for adverse outcomes would have been established and embryos and foetuses showing signs of genetic abnormalities or other abnormal development could be terminated. This means that concerns, for instance, about congenital birth defects (Suter 2016; Notini et al. 2020) from the increased consanguinity involved in solo reproduction can be addressed by embryo screenings so long as the latter too is not objectionable.

Even if people do find preimplantation or prenatal testing objectionable, it is not clear that the biological risk objection would succeed. After all, some legally allowed uses of IVF may in fact be just as risky as solo reproduction even without testing. If it should be legally permitted to perform IVF without preimplantation screening for elderly couples whose gametes have already sustained significant genetic damage (Testa and Harris 2004) or who carry a dominant disease-causing allele like Huntington’s Disease, parity suggests that solo reproduction should not be forbidden either. One reason for this, as Savulescu and Kahane (2009) have argued, is that what matters is their overall quality of life, not whether they have one some medical condition or other. Provided that the child can be expected to have a life which is worth living, the child is not themselves harmed. If they had not been selected (or created), another child would be born. This is an example of “harmless wrongdoing”. So, if people should be permitted to use IVF to create a child with a 50% chance of having Huntington’s disease, they should be permitted to create a solone. This is even more so if the parent intends to use genetic screening to select the child with the best chance of the best life (procreative beneficence).

If, however, solo reproduction is inherently objectionable apart from risk to health, then making the technology reliable will not be enough. With regard to concerns about dignity and the telos of human sexuality, these objections have already been well discussed in the literature. We will discuss each objection briefly and rehearse the replies to those objections to show why they do not work before discussing the vice objection which has garnered less attention in the literature.

### Dignity

The dignity objection is that cloning and solo reproduction violate the dignity of the clone and solone, respectively. However, whether dignity is violated by solo reproduction is going to depend on what is meant by dignity. On some views, dignity, as a concept, should be done away with because it either reduces to respect for the capacity to reason (i.e. Kantian Autonomy), vague restatements of other moral positions or a covert appeal to religious dogma (Macklin 2003; Caulfield 2003). Even defenders of the concept concede that there is significant ambiguity in the usage of the term (Schachter 1983; Malby 2002; Schulman 2008; Rolston 2008). Schulman, for instance, distinguishes between Stoic dignity, Biblical dignity, Kantian autonomy and the accounts of dignity found in the twentieth century international declarations and constitutions (Schulman 2008). Malby distinguishes three strands: one pertaining to Kantian autonomy and the capacity to reason, the second pertaining to a conception of civilised behaviour and the third pertaining to group identity and membership (Malby 2002). Likewise, other commentators have also distinguished between accounts of dignity which relate to respect for Kantian Autonomy and conceptions pertaining to culture and civilisation (Beyleveld and Brownsword 1998; Rolston 2008) or humiliation (Schachter 1983). For our purposes, we can distinguish between two distinct conceptions of dignity: dignity as the capacity to reason and dignity as concern for the preciousness life. We will address each conception in turn.

Consider the first conception of dignity. Concern for dignity as a matter of respect for people’s capacity to reason normally counts against placing restrictions on the choices that they make (Schachter 1983). If that was all to be said about this conception of dignity, then this conception would count in favour of permitting cloning and solo reproduction. However, if one of the options people might take involves failing to respect others’ capacity to reason, that may be grounds to restrict people from choosing that option. Critics of cloning contend that at least some of the purposes with which people might pursue cloning instrumentalises the clone. For instance, if someone were to clone a child in order to replace a dead child or to select perceived favourable characteristics, the thought is that since there is “some intent to control how a new human should be” (Malby 2002, 123), and this uses the clone as a mere means to a personal, selfish end. A parallel argument can also be offered against solo reproduction. While solo reproduction may not likely be used to replace a dead child, it might be used because someone thinks very highly of their own genetic makeup and therefore wishes to produce offspring that is a product only of their own genetic heritage and who would thus emulate themselves as much as possible. Solo reproducing for such a narcissistic reason would, arguably, use the resultant child merely as a means to an end.

Even in the cases where someone pursues solo reproduction for narcissistic or any other objectionable purposes, the argument does not succeed. This is because it contends that the act of cloning or solo reproduction uses the resultant child as a mere means to an end. However, as Caulfield (2003) notes, at the point at which the cloning or solo reproduction takes place, the child does not yet exist. As such, solo reproduction or cloning only treats the gametes or other precursor to the embryo as mere means, not the solone or clone. Moreover, if reproductive acts could use the resultant child merely as a means, then all reproductive acts use the child merely as a means. This would be because children never consent to their own conception (Shiffrin 1999), and violating consent is one of the paradigmatic ways of using someone merely as a means to an end. Yet, few opponents of cloning and solo reproduction are willing to endorse this anti-natalist conclusion (Muralidharan forthcoming).

People may also have children by ARTs like IVF or artificial insemination or by natural means on the basis of objectionable motives. It is not unusual for prospective parents to, at least in part, be motivated by the prospect of having a child that resembles themselves when deciding to pursue IVF rather than adoption. Yet we do not typically find it appropriate to screen potential parents for their motivations.

This brings us to another objection, namely that even if cloning or solo reproduction does not use the resultant child merely as a means, it causes the child to be used merely as a means. For instance, the child’s parent or society may impose unreasonable expectations on the child in virtue of their special origin. This may in turn cause the child to feel like their options are limited. Therefore, or so the objection goes, it is wrong to have children who would be subjected to such harm.

Notably, concerns about instrumentalization and “hyperparenting” (Sandel 2005)) are valid concerns, but they extend to normal reproduction. Many people oppress their children with their expectations of having musical or sporting superstar. If, in a racist society, Black children experience suffering and humiliation for being Black, does it follow that it is wrong to have Black children? Insofar as the suffering someone undergoes is wrongfully inflicted by others, that suffering, plausibly, does not count against the production of such an individual. Likewise, for a child formed from solo reproduction, the harm that arises from the stigma that such a child might face makes the stigmatisation or instrumentalization of that child wrong, not the act of producing the child itself. What is necessary is better parenting, not interference in modes of reproduction. Just as screening for Down syndrome might lead to greater stigmatisation and prejudice towards people with Down syndrome, the response is not to ban Down syndrome screening. The response is to better educate people and provide social support for people with Down syndrome.

Let us now turn to the second conception of dignity. Those who endorse this second conception of dignity suppose that human life, in all its forms, is precious. In particular, even the lives of embryos are extremely valuable. On this view, cloning is wrong because it violates the dignity of the many embryos that are destroyed or discarded in the process of creating the clone (Schulman 2008). Likewise, solo reproduction would also be impermissible because it requires the destruction of embryos, especially if preimplantation genetic screening is used to select for embryos which are unlikely to develop severe birth defects or other genetic diseases. However, views like this prove too much. Such an account of dignity equally condemns IVF which many consider to be acceptable. If we are unwilling to ban IVF, then we should be equally unwilling to ban solo reproduction in the event that it becomes at least as safe and effective as IVF.

### Sexuality and Teleology

Another objection to cloning and solo reproduction is that pursuing such artificial methods of reproduction divorces human sexuality from reproduction (Second Vatican Council 1965; Congregation of the Doctrine of the Faith 1987; Kraynak 2008; Kass 1997). This is supposedly objectionable because the purpose or *telos* of human sexuality is reproduction and divorcing a capacity from its *telos* is wrong.

One problem with this argument is that it proves too much. Procedures like IVF and artificial insemination, which most find unobjectionable, also divorce human sexuality from reproduction. At least some of those who have made the teleology objection against cloning have explicitly considered IVF as objectionable for the same reason (Second Vatican Council 1965; Congregation of the Doctrine of the Faith 1987; Kraynak 2008; Kass 1997; Finnis 1997). Insofar as these procedures should not be banned, the mere fact that cloning and solo reproduction allow people to have children without having sex does not justify banning them.

## Vice, Virtue and the Duty to do Virtuous Things

The focus of this paper is an objection which has received much less attention in the literature. According to this objection, cloning and solo reproduction are typically wrong because they characteristically exemplify and encourage certain vices (Finnis 1997; Kraynak 2008; Sandel 2005). The objection is not that solo reproduction harms, instrumentalises or otherwise wrongs the solone. Rather, it is that even if solo reproduction did not harm or wrong anyone, it would be wrong simply because it expressed certain vices. This, according to the objection, is why solo reproduction should be banned.

Let us first give a plausible rendition of the objection. Cloning and solo reproduction supposedly express and reinforce a kind of Promethean hubris already rife in modern societies because it is an attempt to control the genetic makeup of one’s child. By forgoing more traditional modes of reproduction where the characteristics of one’s child are more left up to chance, parents lose an opportunity to develop a sense of humility and openness to the natural world as given (Sandel 2005).

Some have argued that cloning and, by extension, solo reproduction may be narcissistic in most circumstances (Malby 2002; Kass 1997; Sandel 2005). After all, most motivations to clone oneself involve a certain (excessively) high degree of regard towards oneself and one’s own genetic makeup (Malby 2002).

While there might be some motivations which would be objectionable, there might also be other motivations for cloning and solo reproduction that are not narcissistic. For instance, a woman who has been abused by men all her life may wish to have a child without involving any man even by sperm donation. Or suppose someone has a genetic condition that makes it extremely risky for them to contribute genetically to the offspring. If they and their spouse wish to have offspring without getting genetic material from outside the marriage, they might pursue cloning or solo reproduction if either technology were viable. As earlier mentioned, a prospective single parent may wish to have children without involving the genetic material from outside the family unit in much the same way that couples may wish to have children without involving genetic contribution from outside the nuclear family unit.

Arguably, for some non-narcissistic motivations, other better options are available. For instance, IVG can be used to treat any form of infertility that would have been addressed by cloning and would result in a child equally related to both parents instead of just one. For the woman who has been so traumatised that she does not want to involve any males in reproducing, sperm may be obtained from another woman via IVG (Malby 2002; Sandel 2005).

There are, however, other non-narcissistic motivation for solo reproduction and cloning for which no other ART would suffice. This includes the desire of a single parent to have children while maintaining the genetic integrity of the family unit and the case of the couple where one member has a dangerous mutation that will certainly be passed down. Even if these motivation are not narcissistic, opponents of solo reproduction might regard the desire to asexually reproduce as expressing an objectionable unwillingness to accept one’s place in the world and the limitations placed on us qua human beings (Kraynak 2008; Grisez 1983; Sandel 2005).

At this point, we might object that this is too quick. Cloning and solo reproduction may express a certain degree of self-regard. However, this amounts to hubris and narcissism only if it is excessive. We might then question what makes this excessive. The answer virtue ethicists would give is that these attitudes become excessive and hence vicious only when it hinders others’ or one’s own flourishing (Card 2004). As we have seen so far in the previous section, if the technology actually works, cloning and solo reproduction would not harm or otherwise interfere with the flourishing of others.

There might be one reason for solo reproduction, just as there might be for cloning, which is not narcissistic but benevolent: aiming at maximizing the well-being of the future child. Imagine a person who wishes to have a child genetically related to them who has the best chance of the best life (procreative beneficence). It would be rational to employ as many different modes of reproduction and examine the genes of the embryos by whole genome sequencing to estimate which will start of life with the strongest genetic hand. This would include using partner or donor gametes, clones and solones. It is an open question which would be the best from the perspective of future well-being. It might be a solone. It might be a combination of desire for genetic relatedness (common and unobjectionable) together with genetic advantage that drives solo reproduction.

Nonetheless, many would believe that those who engage in cloning or solo reproduction must believe their genes are in some sense “good” and worth propagating. They are in a sense eugenicists (as are any people who use genetic selection technologies.) Perhaps, then, the thought is that if people clone themselves or engage in solo reproduction, their degree of self-regard and confidence would grow excessively and make it more likely that they will do something that will harm others. One might even reason that the risk of this happening is non-trivial since the people who are already inclined to clone themselves or solo reproduce are already likely to have a high degree of self-regard. It does not follow that solo reproduction is wrong, at least not in the sense we are interested in.

Compare this argument against cloning and solo reproduction with Kant’s argument against mistreating animals. For Kant, we should not mistreat animals because doing so would express cruelty and attenuate our dispositions to feel empathy and sympathy. Attenuating these dispositions would in turn make it more likely treat our fellow humans badly and that would be wrong. For Kant, the duty to not mistreat animals is not directly owed to the animals but is an indirect one owed to oneself (Korsgaard 2018). Given the structural similarity between Kant’s argument for an indirect duty towards animals and the aretaic argument against cloning and solo reproduction, we might note that the duty thus argued for on this picture is a duty owed to oneself.

We might offer a more Aristotelian version of this argument wherein a certain degree of self-regard and confidence becomes excessive when it makes it harder for the possessor of those dispositions to flourish. In this case, the claim would be that parents who choose to solo reproduce supposedly lose out by lacking the wisdom to relate to others with humility and without excessive self-concern. And supposedly, relating to others with humility and without excessive self-concern is constitutive of flourishing. Even here, the duty is owed, ultimately, to oneself, not to others.

There are a number of things we might say against the vice objection. Firstly, consider the claim that engaging in solo reproduction will encourage some vicious disposition that hinders one’s own or others’ flourishing. This claim seems rather speculative. It requires empirical support which has not been provided. In the absence of the encouragement claim, the vice argument would consist of only the claim that solo reproduction expresses a certain vice. However, it would be question begging to suppose that there is any one disposition expressed by solo reproduction, let alone that this disposition is excessive.

Even if opponents of solo reproduction can make good on the encouragement and expressiveness claim, there are still some things we might say. For instance, we might note that we generally do not consider the mere fact that an act expresses or encourages a certain vicious disposition sufficient grounds to coercively restrict the act. For instance, many things are narcissistic or hubristic but still legal (and should remain legal). This would include public boasting and self-aggrandisement, raising children to idolise oneself or to pursue dreams that one may have missed out on and even dating apps which allow one to search for partners who resemble oneself. We also do not consider it appropriate for states to interfere with our choice of recreational activities and vacation destinations even if what we do for recreation can express or even encourage certain vices. For instance, vacationing in Ibiza, a place well known for its club and party scene, may nudge the vacationer towards self-indulgence in sensuous pleasures, while vacationing in Tibet may be more likely to nudge her towards self-reflection. Yet few would think it appropriate for the state to ban certain travel destinations simply because vacationing there would encourage vice. To be sure, while some countries do sharply limit what recreational activities its citizens engage in, this is rarely done for purely aretaic reasons. For instance, drug laws are enacted ostensibly because of concrete harms that widespread drug use may result in. Some extremely religiously conservative countries may restrict behaviour that is seen as sinful or vicious, but these restrictions are widely regarded as illiberal and therefore objectionable.

Even conservatives who oppose cloning like Finnis (1997) deny that the proper role of the state is to make people virtuous. If so, then, in the solo reproduction case, if the only reason to ban solo reproduction would be a concern with vice, it would be inappropriate to ban solo reproduction. We can also offer a more philosophical grounding for why this might be the case.

One problem with at least the more Aristotelian version of the vice argument is that it fails to distinguish between duties which are enforceable and those which are not. According to the vice argument, the duty to not solo reproduce is not owed to anyone else, but only to oneself. However, in order for a duty to be enforceable, that duty must be owed to someone else. After all, if it is owed only to oneself, one could simply choose not to enforce it against oneself. Consider how if you merely promise to yourself and not to anyone else to finish writing a paper by a certain date, you could simply choose to not hold yourself accountable if you miss your own personal deadline. By contrast, if you break a promise you make to another, they are within their rights to hold you accountable and you cannot unilaterally absolve yourself from accountability. This is because when someone owes us a duty, that gives us standing to hold them accountable. That is just what it means for us to have a claim on someone else.

Moreover, it would be wrong for us to coerce people to do something if they are not accountable to anyone (apart from themselves) for not doing it. After all, part of what it means for one person, A, to be accountable to B for doing something, ф, is that B has a claim on A that she ф. Coercing someone to ф when we do not have a claim against them that they ф (i.e. when they do not owe it to us and hence are not accountable to us to ф) and there is no one on whose behalf we are acting who has such a claim is arguably authoritarian (Mill 1895, 139). This is because it is inconsistent with treating one another as moral equals.

However, on the Aristotelian account, a person’s hubris is bad only because it makes her excessively dissatisfied with her own human frailty and limitations or otherwise leads her to do other things that would inhibit her flourishing. Likewise, narcissism is bad because it prevents people from relating in the right way with others and so hinders the formation and health of important relationships (Sandel 2005). To generalise, for any other disposition, it counts as a vice because it impedes the agent’s own flourishing. As such, on the Aristotelian account, there is no one else to whom the agent is accountable for exercising and developing the virtues and avoiding vice. It would therefore be wrong to ban solo reproduction even if, in the given context, solo reproduction would express some vice and even encourage vice.

At this point, one might object that we are too quick. Supposedly, our argument presupposes an excessively individualistic conception of virtue. It seems perfectly acceptable to interfere with other people’s self-destructive decisions in some cases. For instance, when someone’s loved one is engaged in self-destructive behaviour, it seems appropriate to stage an intervention wherein participants invoke the fact that they care for the troubled individual and are intervening out of concern (and not judgement). If interventions of these sort are permissible, and perhaps sometimes even required, then other people can have claims on us to promote our own flourishing.

However, just because some people have a claim on us for us to act for our own benefit, it does not follow that the state has any such claim. Consider that in an intervention, the only people who have a claim are those who are close friends and family members of the troubled individual. It is considered inappropriate for participants in an intervention to bring along someone who is not close to the troubled individual. This is at least partly because they lack the standing to make a claim on the troubled individual. The unrelated individual, unlike the close friend or relative, cannot call upon the closeness of their relationship to justify their paternalistic intervention. If this is right, then a thick relationship between two people is required in order for one person to have any paternalistic claim on the other. And even then, perhaps only if there is significant risk of serious harm. However, the relationship between two citizens who are otherwise merely strangers to each other or between the state and its citizens is not that thick.

A related objection is that perhaps there are other kinds of virtue theories (e.g. Kantian ones) wherein possessing the virtues is not just good for one’s own flourishing. After all, if a person had a hubristic or narcissistic disposition, might she not be more disposed to wrong others? Perhaps a sufficiently narcissistic person might, because of some perceived slight, decide to run for public office and, if successful, cause lots of harm. Since people have a claim against others wronging them, they should also have a claim against others being hubristic or narcissistic. That is to say, not being hubristic or narcissistic is not just an impersonal duty but is something that is owed to those we might wrong by having such vicious dispositions.

However, while people do have a claim against other people being narcissistic or hubristic, that does not entail that they have a claim against them performing any narcissistic or hubristic action at all. Consider that solo reproduction is neither necessary nor sufficient for hubris and narcissism. Even without solo reproduction, there are plenty of narcissists and arrogant people around. Neither is it plausible that everybody who engages in solo reproduction will become narcissistic or hubristic. It is implausible that any one action has such immense effects on our dispositions. Rather, the most plausible version of the encouragement claim is merely that someone who pursues solo reproduction may increase their disposition to be self-regarding. Where this disposition becomes excessive, they become narcissistic. Someone who solo reproduces may refrain from other actions that might increase their self-regard and perform other actions that increase their humility as well as their other-regarding-ness. This means that while we may owe it to others to not neglect our moral character to such an extent that we become vicious, people have no claim on our performing or not performing any given action that would shape our moral character in small ways.

If this is right, then the vice argument does not work. It would be wrong to ban solo reproduction simply because it encourages certain vices.

## Conclusion

As we have seen so far, the vice argument is not successful because no matter how we spell out our account of virtue and vice, the mere fact that solo reproduction might encourage certain vices does not given anyone else a claim on a solo reproducer to desist from this activity. What is needed is the further claim that solo reproduction or cloning wrongs or harms someone in certain concrete ways. Yet, we have also seen that many of the arguments that solo reproduction would harm or wrong others are either unsuccessful or depend on certain religious or otherwise controversial account of the good life. If this is right, then neither solo reproduction nor cloning should be banned if the technology became no more physiologically risky than all other currently accepted ARTs.

If people have a right against interference with their solo reproduction, then they must also have a right against interference with other uses of IVG. After all, any objection that might be made against using IVG to treat infertility of different-sex couples, help same-sex couples or transgendered individuals would also apply to using IVG for solo reproduction. Worries about dignity, the telos of human sexuality and hubris may still be raised against these other uses of the technology. Since the objections are insufficient to make restrictions on solo reproduction legitimate, neither would any objections to the other uses of IVG.

There might of course be other concerns about harms to innocent third parties like surrogates or distributive justice concerns relating to whether solo reproduction may be the best use of scarce resources. However, these concerns only require us to be careful in how solo reproduction is carried out and whether and how it should be subsidised, not whether it should be permitted at all. Solo reproduction, insofar as the technology becomes sufficiently safe and reliable, would be no worse than IVF and other legally permitted ARTs on this score.

What should states do? Firstly, there should be no permanent ban on solo reproduction as such. Countries like the UK which permit reproduction using only naturally generated gametes should repeal or modify those laws. However, this does not mean that solo reproduction should be permitted right now. The procedure, as it stands, is still extremely risky. Instead, two kinds of policy options suggest themselves.

Ideally, if a state already has general regulations about how risky reproductive technologies can be (as Singapore does), those regulations should be extended to cover (if they do not already do so) solo reproduction and other IVG-based methods. Due to increased consanguinity, the safety and reliability of solo reproduction would have to be assessed separately from other uses of IVG technology. Nevertheless, the same safety standards should apply to all reproductive technologies. In the absence of such safety regulations, states should enact such laws.

Less ideal would be a temporary moratorium on solo reproduction which could be renewed every few years. However, status quo bias on the part of legislators may result in legislators periodically renewing the ban even when the technology is sufficiently safe and reliable.

## Data Availability

NA.

## Declarations

1 Each of us has two copies of any given gene. Let us call the copies of some gene of a person, Sarah, A and B. There is a 50% chance any given sperm cell generated from Sarah will have gene A and 50% chance it will have B instead. Likewise, there is a 50% chance that her egg will have A and 50% chance it will have B. If she solo reproduces, there is a 25% chance that her daughter will inherit 2 copies of A, 50% chance she will inherit one copy each of A and B and 25% chance she will inherit 2 copies of B. This means that there is a 75% chance she will inherit at least one copy of A and also a 75% chance she will inherit at least one copy of B.

2 In Bradford, UK, where marriage between first cousins is common, the base rate of severe birth defects is about 3%, while it goes up to 6% for the offspring of first cousins (Archives of Disease in Childhood 2014). Meanwhile, the base rate of severe birth defects for the UK is 1%.

3 Let us assume, conservatively, that the likelihood of severe birth defects for solo reproduction would be quadruple that of parent–child or sibling incest. If the base rate is 1%, incest increases the risk by 6 × and solo reproduction increases it by a further 4×, we can expect the risk to be 24% for someone with a typical genetic load. This risk increases if, for some reason or other, there are more deleterious recessive mutations in the individual. This is roughly what we would expect if the parent had just one severe recessive genetic defect.
